# HER2 Testing Characteristics Can Predict Residual Cancer Burden following Neoadjuvant Chemotherapy in HER2-Positive Breast Cancer

**DOI:** 10.1155/2021/6684629

**Published:** 2021-05-24

**Authors:** Tamera J. Lillemoe, Mara Rendi, Michaela L. Tsai, Monica Knaack, Rina Yarosh, Erin Grimm, Barbara Susnik, Janet Krueger, Susan Olet, Karen K. Swenson

**Affiliations:** ^1^Allina Health Laboratory-Hospital Pathology Associates, 800 East 28th Street, Minneapolis, MN 55407, USA; ^2^Allina Health System, Virginia Piper Cancer Institute Clinical Research Program, 800 East 28th Street, Minneapolis, MN 55407, USA; ^3^Allina Health System, Research Informatics, 925 Chicago Avenue, Minneapolis, MN 55407, USA

## Abstract

**Objectives:**

The response to HER2-targeted neoadjuvant chemotherapy (NAC) in HER2-positive (+) breast cancer can be quantified using residual cancer burden (RCB) pathologic evaluation to predict relapse free/overall survival. However, more information is needed to characterize the relationship between patterns of HER2 testing results and response to NAC. We evaluated clinicopathologic characteristics associated with RCB categories in HER2+ patients who underwent HER2-directed NAC.

**Methods:**

A retrospective chart review was conducted with Stage I–III HER2+ breast cancer cases following NAC and surgical resection. HER2 immunohistochemistry (IHC) staining and fluorescence in situ hybridization (FISH), histologic/clinical characteristics, hormone receptor status, and RCB scores (RCB-0, RCB-I, RCB-II, and RCB-III) were evaluated.

**Results:**

64/151 (42.4%) patients with HER2+ disease had pathologic complete response (pCR). Tumors with suboptimal response (RCB-II and RCB-III) were more likely to demonstrate less than 100% HER2 IHC 3+ staining (*p* < 0.0001), lower HER2 FISH copies (*p* < 0.0001), and lower HER2/CEP17 ratios (*p* = 0.0015) compared to RCB-I and RCB-II responses. Estrogen receptor classification using ≥10% versus ≥1% staining showed greater association with higher RCB categories.

**Conclusions:**

HER2+ characteristics show differing response to therapy despite all being categorized as positive; tumors with less than 100% IHC 3+ staining, lower HER2 FISH copies, and lower HER2/CEP17 ratios resulted in higher RCB scores.

## 1. Background

Neoadjuvant chemotherapy (NAC) has historically been administered to breast cancer patients presenting with more advanced disease stages to reduce tumor size prior to surgery, rendering previously inoperable tumors operable, and potentially making breast conserving surgery a more viable option. More recently, NAC has been used for earlier stage disease to provide an objective measure of response to treatment, and to more rapidly assess novel therapies in clinical trials. Many studies have shown that pathological complete response (pCR) after HER2-directed NAC is predictive of recurrence-free survival in HER2+ breast cancers; [1–5] however, a significant number of patients have residual tumor following NAC. The residual cancer burden (RCB) index was developed by MD Anderson Cancer Center to quantify residual disease upon pathological review and predict survival after NAC for breast cancer [6–8]. The RCB index incorporates the remaining post-NAC primary tumor dimensions, cellularity of the tumor bed, in situ cancer remaining within the tumor bed, number of involved lymph nodes, and size of largest axillary metastasis.

The importance of molecular tumor profiles in predicting treatment response to NAC has been previously reported. Patients with hormone receptor-negative breast cancer derived the greatest benefit from neoadjuvant chemotherapy [9]. In 2013, Lee et al. found that in a study of 345 breast cancer patients, estrogen (ER) and progesterone (PgR) positivity and tumor size were inversely associated with pCR; HER2+ and triple-negative tumors were more likely to achieve pCR than hormone receptor-positive and HER2-negative cancers [10]. Recent meta-analyses of eleven HER2+ neoadjuvant clinical trials found that pCR was associated with smaller tumors that were hormone receptor negative [11]. Ki-67 index, a marker of cell proliferation, has also been found to be an independent predictor of pCR when measured by objective quantitative methods [12]. Other studies have confirmed that molecular subtype (ER and PgR expression and HER2 status) and Ki-67 index were significant independent predictors of pCR and disease-free survival [13–15]. Increased levels of stromal tumor-infiltrating lymphocytes (TILs) on prechemotherapy biopsy also have predicted higher rates of pCR response to NAC, particularly in HER2+ or triple-negative disease [16]. In addition, dual HER2-directed chemotherapy versus single-agent therapy has also been shown to improve pCR rates in the neoadjuvant setting. The NeoSphere Study found that neoadjuvant docetaxel + pertuzumab/trastuzumab significantly improved pCR compared to docetaxel + trastuzumab alone, with similar toxicity [17]. Similarly, in the adjuvant setting, the Aphinity trial found that the addition of pertuzumab to trastuzumab + chemotherapy significantly improved disease-free survival [18].

The determination of HER2 status is based on guidelines published by the American Society of Clinical Oncology (ASCO) and the College of American Pathologists (CAP). The guidelines published in 2013 [19] were further redefined in 2018 [20], incorporating data from Press et al. [21–22] HER2+ tumors are identified by immunohistochemistry (IHC), fluorescence in situ hybridization (FISH), or a combination of these testing modalities. A small percentage of tumors require both IHC and FISH results to determine overall HER2 status. Studies have found that the level of HER2 amplification, in particular the HER2 gene copy number, has been associated with response and survival following HER2-directed NAC.^15^ [23–24], However, more studies are needed to determine detailed HER2-specific testing results and correlation with recurrence and survival outcomes after NAC.

This study was conducted to further refine clinicopathologic features associated with extent of residual disease following NAC as measured by RCB index in patients with HER2+ disease identified on immunohistochemistry (IHC) and/or FISH analyses. The findings from this analysis will be used to determine if there are certain pretreatment characteristics of HER2-positive disease that predict RCB following HER2-directed NAC.

## 2. Methods

This investigation was a retrospective chart and pathology review of patients diagnosed with HER2+ breast cancer who had HER2-directed NAC at the Allina Health System from January 1, 2013 to June 30, 2017. The Allina Health Institutional Review Board approved this project as an expedited study. Cases were selected from the pathology database with the following inclusion criteria: [1] Stage I–III HER2+ breast cancer diagnosed at the Allina Health System, [2] receipt of HER2-directed NAC, [3] surgical resection following NAC at an affiliated hospital, and [4] patient release of medical records for research. Consecutive cases meeting these criteria were included in this review. Original HER2 status (either IHC or FISH) defined based on the ASCO CAP 2013 published guidelines [19] and hormone receptors using image analysis (Aperio Image Analysis System) were interpreted prior to NAC in the Allina Health Laboratory by one of the authors (TJL, EG, and BS). Alternative HER2 testing was retrospectively performed on the original core biopsies on available paraffin blocks and interpreted by the pathology authors. Additional HER2 IHC review was performed by one of the authors (TJL) to determine the overall estimated percentage of tumor cells with 3+ staining. All studies were performed and analyzed at Allina Health Laboratory (FISH testing using the PathVysion HER2 DNA Probe Kit by Abbott Molecular, Inc. (according to the package insert), or IHC using the Ventana anti-HER2/neu-monoclonal antibody (4B5) on a Ventana BenchMark Ultra immunostainer). Image analysis was not performed for HER2 IHC.

Each HER2-positive tumor was also ultimately evaluated based on the updated 2018 American Society of Clinical Oncology (ASCO)/College of American Pathologists (CAP) Clinical Practice Guidelines for comparison [20].

These ASCO/CAP guidelines categorize HER2 FISH results into 5 groups (Table 1) based on HER2/CEP17 ratio and average HER2 copy number/cell:

The HER2 IHC guidelines were slightly modified in 2018 as follows:

1. IHC 3+ positive: circumferential complete, intense membrane staining in >10% of tumor cells
2. IHC 2+ equivocal: weak to moderate complete membrane staining in >10% of tumor cells, moderate to intense but incomplete (basolateral or lateral) staining, or circumferential intense staining in ≤10% of cells
3. IHC 1+ negative: incomplete membrane staining that is fain/barely perceptible in >10% of tumor cells
4. IHC 0 negative: no staining or incomplete, faint/barely perceptible staining in ≤10% of tumor cells

### 2.1. Data Collection

Chart review was conducted to gather treatment and outcome data. Information collected included HER2 testing results (HER2 IHC category and percentage of 3+ staining, HER2/CEP17 ratio, and HER2 and CEP17 absolute copies), and HER2 heterogeneity if present (by either IHC and or FISH). We also collected histologic and demographic characteristics including ER and PgR status, tumor size and nodal status pre- and postchemotherapy, presence of tumor-infiltrating lymphocytes (TILs), and patient age at diagnosis.

Three authors (TJL, EG, and BS) reviewed the histologic slides from each surgical specimen and calculated the RCB index to quantify RCB following neoadjuvant treatment. Neoadjuvant chemotherapy treatment patterns were described by treatment regimen and dual- versus single-agent HER2-directed therapy. Follow-up time was determined for each subject from the time of diagnosis to the most recent follow-up visit.

### 2.2. Data Analyses

The data are presented as counts and proportions for categorical variables while mean ± standard deviation is used for continuous variables. A comparison of neoadjuvant chemotherapy (RCB-0, RCB-I, RCB-II, and RCB-III) categories was done using a nonparametric approach, the Kruskal-Wallis test. Further analysis entailed determining predictors of residual cancer burden (RCB-0, RCB-I, RCB-II, and RCB-III) as an ordinal outcome by fitting univariate and multivariate logistic regression models. PROC LOGISTIC in SAS version 9.4, analytical product SAS/STAT 15.1 fitted the proportional odds model to the ordinal outcome. Association of potential predictors/factors (HER2/CEP 17 ratio, HER2 absolute copy numbers, HER2 IHC category and percentage of staining, tumor grade, clinical stage, patient age, ER status, PgR status, and hormone sensitivity) with RCB after HER2-directed NAC was examined using odds ratios (OR) with their associated 95% confidence limits. Treatment groups were categorized according to anthracycline versus taxane-based neoadjuvant chemotherapy, and dual- versus single-agent HER2-directed therapy and compared by RCB category. All tests were done at a 5% level of significance, and statistical analysis was performed using SAS Version 9.4, analytical product SAS/STAT 15.1.

## 3. Results

Between 2013 and 2017, 151 patients were diagnosed with HER2+ breast cancer (based on IHC or FISH) and treated with HER2-directed NAC at an Allina-affiliated facility. Characteristics of the patients in each RCB category are listed in Table 2. The predominant tumor type was invasive ductal, NOS (134 cases); 13 of these cases showed at least 50% tumor TILs. Eleven cases were invasive micropapillary, and there was a single case each of mucinous, mixed ductal-lobular, and apocrine. Three cases were invasive lobular (1 of which was pleomorphic type). At the time of surgical resection, 64 patients (42.4%) had a pCR (RCB-0). Eighty-seven patients had residual tumor; 32 cases (21.2%) were classified as RCB-I, 47 cases (31.1%) as RCB-II, and 8 cases (5.3%) as RCB-III following NAC.

Dual HER2 IHC and HER2 FISH results were performed on 150/151 tumors (Table 3). The correlation between HER2 IHC and FISH testing modalities was 97% (146/150). Four tumors exhibited disparate HER2 results when comparing the testing modalities. Two cases in FISH Group IA showed HER2 IHC 0 staining, and following HER2-directed NAC, they had residual disease classified as RCB-I and RCB-II. Two cases in FISH Group 5 showed HER2 IHC 3+ staining of 60% and 15% and were classified as RCB-II and RCB-III, respectively.

**Figure 1:**
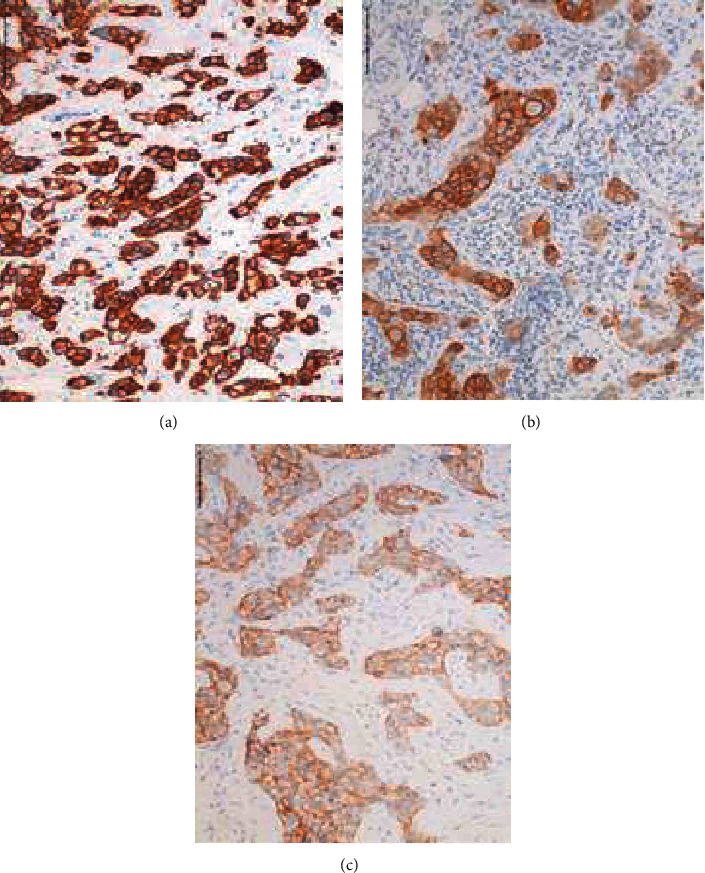
Patterns of HER2 IHC staining: (a) 100% HER2 IHC staining; (b) >10% and <100% HER2 IHC staining; (c) HER2 IHC 2+ staining.

Overall, cases in the RCB-II and RCB-III categories showed different HER2 profiles by both IHC and FISH compared to the RCB-0 and RCB-I categories. When evaluating IHC HER2 testing, there were different degrees of HER2 staining. Tumors with >10% of tumor cells showing strong circumferential staining were classified as 3+. However, within the 3+ staining category, some tumors exhibited nearly 100% of tumor cells with strong staining (Figure 1(a)), while other tumors contained obvious areas lacking strong staining (>10% but <100%) (Figure 1(b)). Tumors scored as 2+ showed <10% of tumor cells with strong circumferential staining (Figure 1(c)).

**Figure 2:**
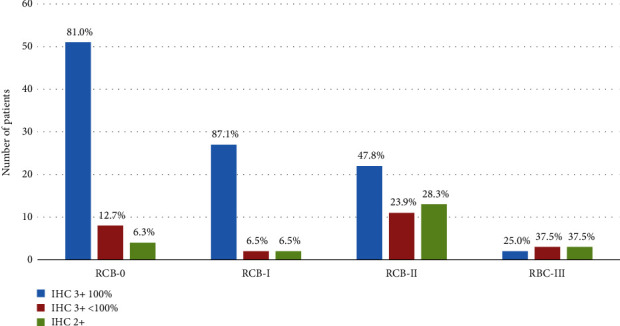
HER2 IHC staining characteristics by post-neoadjuvant residual cancer burden categories.

There was a significantly different distribution of HER2 IHC staining categories based on RCB score (*p* < 0.0001) (Figure 2). The majority of cases (81.0%) with a pCR (RCB-0) exhibited virtually 100% 3+ HER2 expression compared to those tumors with IHC 3+ <100% expression (12.7%) and IHC 2+ (6.3%) categories. There was a similar pattern in patients with residual tumor classified as RCB-I; the majority of cases exhibited almost 100% 3+ HER2 expression (87.1%) compared to those tumors with <IHC 3+ <100% expression (6.5%) and IHC 2+ (6.5%).

**Figure 3:**
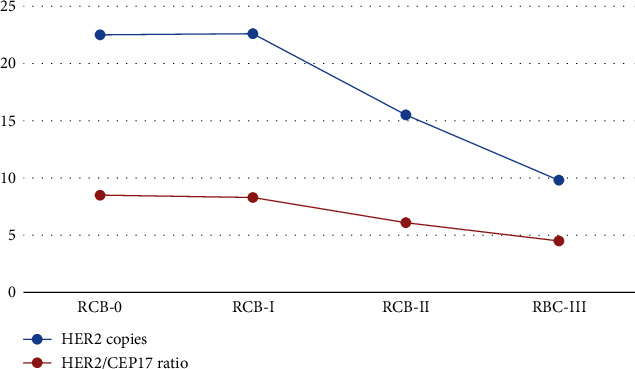
Mean HER2 copies and HER2/CEP17 ratios by post-neoadjuvant treatment residual cancer burden categories.

When using FISH to determine HER2 status, RCB-II and RCB-III cases had significantly fewer mean HER2 copies (15.5 and 9.8, respectively) compared with RCB-0 and RCB-I cases (HER2 copies 22.5 and 22.6, respectively, *p* < 0.0001) (Figure 3). RCB-II and RCB-III cases also had significantly lower HER2/CEP17 ratios than RCB-0 and RCB-I cases (6.1 and 4.5 for RCB-II and RCB-III versus 8.5 and 8.3 for RCB-0 and RCB-I, *p* = 0.0015).

**Figure 4:**
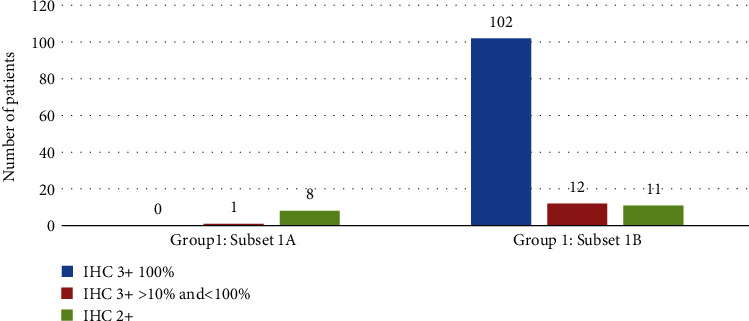
HER2 IHC staining characteristics of FISH Group 1 Subset A and Subset B.

Using the 2018 ASCO/CAP Guidelines, cases in HER2 testing Group 1 comprised the vast majority of our cases (88.7%; 134/151 patients). However, this category showed differences in extent of residual disease post-NAC compared to original HER2 gene copy numbers and overexpression of HER2 by IHC. Thus, this category was further classified into Subsets 1A (≥4 to <6 HER2 copies per cell) and 1B (≥6 copies per cell). Upon final analysis, only 22.2% of tumors in Subset 1A had RCB-0 or RCB-1 responses compared to 71.2% of those patients in Subset 1B (Figure 4). None of the nine tumors in Subset 1A showed 100% circumferential 3+ HER2 IHC staining (only one exhibited 3+ staining in some of the cells) (Figure 4). In contrast, tumors in Subset 1B (*n* = 125) were more likely to exhibit virtually 100% circumferential 3+ expression (81.6%) rather than less than 100% 3+ expression (9.6%).

There was only one case in Group 2 in our database with the 2013 ASCO/CAP guidelines in place at the time of diagnosis (0.67%). This case would not qualify as HER2+ based on the 2018 updated ASCO/CAP guidelines (2+ HER2 IHC staining), but it exhibited residual tumor categorized as RCB-I. In Groups 3 and 4, only 28.6% (4 of 14 cases) had a pCR (RCB-0); the remainder had significant residual disease (71.4% in RCB-II or RCB-III). According to the 2018 updated HER2 guidelines, tumors in Group 4 would now be classified as HER2 negative unless the HER2 IHC score was 3+. We had 7 cases in the Group 4 FISH category, one of which exhibited IHC 2+ staining. The remaining 6 cases all demonstrated 3+ staining in >10% and <100% of cells and are considered HER2 positive based on the current guidelines. Although the numbers are small, most of the Group 4 cases showed significant tumor burden (5 RCB-II) following HER2-directed therapy. In the Group 5 FISH category, there were 2 cases (both of which originally showed 3+ HER2 IHC staining); these 2 cases had residual tumor classified as RCB-II and RCB-III.

There were two cases with HER2 tumor heterogeneity prior to HER2-directed NAC. One case (Group 5) showed differing results between the original core biopsies of the breast cancer (less than 10% HER2-positive cells by both IHC and FISH) and the metastatic disease in the lymph node (>10% HER2-positive cells by both IHC and FISH). There were no histologic differences between these areas. The second tumor was in Group 4 and had one cluster of HER2 amplified cells which comprised approximately 25% of the total tumor on core biopsy (HER2/CEP17 ratio of 1.43, mean HER2 of 6.7, with less than 10% of the cluster cells showing 3+ staining). This clustered population was histologically distinct from the nonamplified tumor. Both of these cases had residual tumor burden categorized as RCB-II following HER2-directed NAC.

We also evaluated the response by hormone status (ER and PgR) using both a ≥1% cutoff and a ≥10% cutoff to define hormone positivity. When using 1% ER staining as a cutoff, there was a twofold increase in pCR/RCB-0 responses in ER-negative tumors compared to ER-positive tumors (*p* = 0.0304). However, when ER positivity was defined as ≥10% staining, there was a threefold increase in pCR/RCB-0 responses in ER-negative tumors compared with ER-positive tumors (*p* = 0.0022). As the ≥10% staining benchmark has a stronger association with higher RCB scores and is often used in clinical situations when determining the best course of treatment for a patient, we included this definition of hormone sensitivity in further analyses.

We evaluated the extent of residual disease based on a neoadjuvant regimen and found no significant differences between regimens. Of patients receiving taxane-based NAC, 67% had RCB-0 and RCB-1, compared to 55% of patients receiving anthracycline-based NAC. Forty-eight (31.8%) patients received a single HER2-directed therapy (trastuzumab), and 103 (68.2%) received a dual HER2-directed therapy (trastuzumab and pertuzumab). The dual therapy resulted in 46% of patients with a pCR (RCB-0), while the single therapy showed 35% of patients with a pCR (RCB-0), although the response difference between the two groups was not statistically significant.

Significant predictors of RCB on univariate analysis included all three measures of HER2 status: HER2 IHC category (*p* < 0.0001), HER2 copy number (*p* < 0.0001), and HER2/CEP17 ratio (*p* < 0.0142) (Table 4). Other predictors associated with lower RCB on univariate analyses included lower pretreatment clinical stage (*p* = 0.0246), smaller pretreatment tumor size (*p* = 0.0142), and hormone-insensitive tumors (*p* = 0.0046).

Several multivariate models were fitted to determine predictors of RCB categories (0, I, II, and III).  Table 5 shows the three predictive models for identifying RCB scores in our patient population with the HER2 FISH mean copy number (Model 1), the HER2/CEP17 ratio (Model 2), and the HER2 IHC categories (Model 3). When evaluating the HER2 mean copy number in the multivariate model, significant predictors of RCB included the HER2 mean copies (*p* = 0.0107), hormone insensitivity (*p* = 0.0012), and pretreatment tumor size (*p* = 0.0060). When evaluating the HER2/CEP17 ratio, significant predictors of RCB included the HER2/CEP17 ratio (*p* < 0.0001), hormone insensitivity (*p* = 0.0016), and pretreatment tumor size (*p* = 0.0084). With the model evaluating the HER2 IHC expression level and staining percentage (Model 3), significant predictors of RCB included the IHC expression level (IHC 2+ vs. 3+), and the percentage of IHC 3+ cells staining positive (100% vs. <100% but >10%) (*p* < 0.0001), hormone insensitivity (*p* = 0.0006), and pretreatment tumor size (*p* = 0.0028). These models demonstrate that each of these methods of determining HER2 status are predictive of RCB score. Model 3 was the most parsimonious model with a c-statistic of 0.721, indicating a high level of prediction of RCB when evaluating the factors of HER2 IHC expression and IHC staining percentage of 3+ 100% vs. 2+, (OR = 7.59 (2.98 − 19.35); *p* < 0.0001), hormone sensitivity (OR = 3.19 (1.64 − 6.2); *p* = 0.0006), and pretreatment tumor size (*p* = 0.0028).

At a mean follow-up time of 35.2 months, overall survival for the entire patient group was 97.3% (146/150 patients alive with 1 patient lost to follow-up). Recurrences were significantly less likely in the RCB-0 category (4.7%) and the RCB-I category (6.3%) than in the RCB-II category (14.9%) and the RCB-III category (37.5%) (*p* = 0.018). Of the four patients who were deceased secondary to breast cancer at the most recent follow-up, there was one patient from the RCB-0 category (1.6%), two patients from the RCB-II category (4.3%), and one patient from the RCB-III category (12.5%). These numbers were too small to conduct meaningful statistical analysis of survival.

## 4. Discussion

The determination of HER2 status in invasive breast cancer is crucial in deciding treatment modalities and in predicting breast cancer treatment response and survival. Although the HER2 IHC or FISH results are often viewed as binary (positive or negative), in reality, there is a spectrum of HER2 results inherent in both testing methods. Our study shows that in the HER2-positive group of tumors, the degree of HER2 positivity by IHC staining and FISH copy number/ratio are both correlated with response to neoadjuvant HER2-directed chemotherapy. Consequently, our data can help clinicians better predict not only the response to chemotherapy but also the overall prognosis of the patient based on the degree of HER2 positivity. As there are limited studies addressing this underlying biology, a major goal of this research was to correlate NAC outcomes with HER2 testing results (using both IHC and FISH studies) to further our understanding of the spectrum of HER2 results.

Recent updates to ASCO CAP HER2 guidelines have improved testing interpretation and clarity for pathologists to determine which patients may benefit from HER2-directed chemotherapy [19–20]. Data reported by Krystel-Whittemore et al. in 2019 [25] and by Meisel et al. in 2020 [15] illustrated that tumors with a pCR are more likely to exhibit HER2 IHC 3+ staining (compared to 2+) in HER2 FISH-amplified tumors. In 2016, studies performed by Press et al. have also shown that HER2 3+ staining correlates with HER2 FISH categories and outcomes [21]. However, previously reported studies have not correlated the relative percentage of HER2 IHC 3+ staining in a tumor with HER2 FISH results and outcomes.

Our findings illustrate that the amount of 3+ staining is significant in predicting residual tumor burden following NAC. HER2-positive tumors that exhibited less than 100% circumferential and strong 3+ IHC staining were consistently associated with greater residual tumor burden after HER2-directed NAC. Although HER2 overexpression by IHC is defined by ASCO/CAP as >10% complete, circumferential, and strong IHC 3+ staining, there are currently no published studies correlating the significance of the relative amount of 3+ staining in a tumor with response to NAC.

The analysis of our cases reveals statistically lower residual tumor burden when tumors exhibited 100% IHC 3+ staining versus less amounts of 3+ staining (>10% to <100%). This staining assessment is relatively easy to appreciate microscopically as tumors with 3+ overexpression either show nearly all cells with 3+ staining compared with tumors that, while also categorized as 3+, show obvious areas lacking uniform, circumferential, strong, and complete staining. In addition, CAP protocols for reporting HER2 staining require that the relative percentage of 3+ staining be recorded, so this information can be easily obtained from synoptic CAP reporting of HER2 IHC. The prognosis of patients with >10%, but <100% IHC 3+ staining is significantly poorer than those with 100% IHC 3+ staining, providing useful prognostic information that should be conveyed to the treatment team.

Our data further support that the degree of HER2 FISH absolute copy numbers and ratios correlate with residual tumor burden following NAC, and that there is a direct correlation with IHC staining. In our analysis, tumors with lower mean HER2 copies and ratios were statistically less likely to have RCB-0 and RCB-I results following NAC as compared with tumors with higher mean HER2 copies, similar to data published by Meisel et al. [15] Results from biomarker analysis of the APHINITY trial presented at the 2019 ASCO meeting showed that tumors in Group 1 with HER2 copy number ≥ 6 had significantly better prognosis and greater benefit from pertuzumab therapy [26]. However, based on our results, we are able to further separate tumors in Group 1 and consequently better predict RCB and overall prognosis following NAC. Tumors in Group 1 with ≥4 to <6 HER2 copies/cell were defined as Subset 1A, while those with ≥6 HER2 copies/cell were defined as Subset 1B. Tumors in Subset 1A showed significantly less benefit from HER2-directed NAC than those in Subset 1B even though all cases are categorized as HER2 positive per guidelines. These data provide important prognostic information that can inform future therapy.

The correlation of clinical outcomes with HER2 testing results was initially reported by Press et al. in 2016 [21], for patients who underwent HER2-directed NAC based on testing guidelines published in 2013 [19]. They utilized retrospective data from 4,269 patients enrolled in the Breast Cancer International Research Group- (BCIRG-) 005, BCIRG-006, and BCIRG-007 clinical trials. Only Group 1 tumors significantly correlated with HER2 overexpression by IHC; these tumors were more likely to show 2+ to 3+ IHC overexpression compared to Groups 2, 4, and 5 (Group 3 exhibited a mixed pattern of overexpression). Tumors in Group 1 showed greater benefit from HER2-directed NAC compared to other groups. Correlations between HER2 FISH groups and IHC overexpression were further reported by Press et al. in an analysis of 7,526 breast cancers in their consultation service [22].

Clinical outcomes have been less understood in tumors that exhibit amplification in the centromere region as well as in the HER2 gene. In tumors with ≥6 HER2 copies but HER2/CEP17 ratios less than 2 (Group 3 patients), the IHC staining pattern is used to determine the overall HER2 status (based on current guidelines for Group 3, 2+ or 3+ staining is considered HER2 positive). Previous data have shown two emerging patterns: the majority of tumors show no HER2 overexpression, and only a minority show 3+ HER2 IHC staining. Most patients in this category have been reported to show limited response to HER2-directed NAC [21–22]. In our study, almost all tumors in this FISH group showed either 2+ staining or <100% 3+ staining. The majority of patients had significant tumor burden following NAC despite ≥6 HER2 copies per cell (RCB scores of RCB-II or RCB-III). Thus, the response in this group of patients was mixed, as also reported by Press et al. [21] As a result, it appears that coamplification of both the HER2 gene (at least 6 HER2 copies) and the centromere region may not portend to improved outcomes with HER2-directed NAC compared to those in Subset 1B.

Emerging evidence of tumor heterogeneity for HER2 expression has been reported in up to 40% of breast cancers [27–28]. In 2017, Hou et al. evaluated tumors with incomplete treatment response to HER2-directed NAC; HER2 heterogeneity was identified more frequently in these cases compared to those who had a pCR [29] Although we did not evaluate tumors for HER2 heterogeneity after NAC, both patients in our study with known tumor heterogeneity before NAC had significant disease burden (RCB-II) after HER2-directed NAC.

Our results also demonstrate a stronger association between hormone receptor-negative tumors and reduced extent of residual disease (RCB-0 and RCB-I) when hormone receptor negativity was defined as <10% rather than <1%. Although CAP guidelines published in 2010 modified the definition of hormone receptor positivity to <1% for treatment purposes [30], other studies have also shown greater reduction in residual tumor burden after HER2-targeted therapy when <10% vs. <1% was used to define hormone receptor negativity and have led to the formation of the low-positive ER group [31]. Our data show that tumors with ≥10% ER expression combined with low HER2 FISH ratios (≥2 to <5) are less likely to respond to HER2-directed NAC. Similar results have been seen with triple-negative tumors; recent studies have shown that HER2-negative breast cancers with ER expression < 10% behave clinically like triple-negative tumors in terms of pCR and survival outcomes [32]. The recent 2020 ASCO/CAP updates by Allison et al. further support that tumors with <10% estrogen receptor staining may derive limited benefit from adjuvant hormonal therapy and may be more effectively treated as triple-negative cancers [33]. Our data indicate that ER < 10% may also be a more clinically important distinction than ER < 1% in HER2-positive tumors as the 10% cutoff more accurately predicts response to HER2-directed NAC.

In summary, our data illustrate the importance of the relative percentage of protein overexpression (HER2 IHC staining) in conjunction with mean HER2 copies and HER2 ratios in predicting RCB and prognosis. Our study is the first to recognize that tumors with <100% and >10% IHC 3+ staining that are nonetheless categorized as HER2 positive based on current ASCO/CAP guidelines are less likely to achieve a pCR and more likely to have higher RCB scores than those with 100% IHC 3+ staining. Further study is needed to confirm this finding in a larger patient population. Analysis of several multivariate models to predict RCB including the HER2 IHC category (IHC 3+ 100% staining), the HER2 mean copy number, and the HER2/CEP17 ratio clearly demonstrated that the model using the HER2 IHC expression and staining percentage was the best predictor of RCB. However, the HER2 mean copy number and the HER2/CEP17 ratio also significantly predicted RCB after HER2-directed NAC, as expected. Other significant predictors of improved RCB scores in both models included hormone-insensitive tumors (0-<10% of cells positive for hormone receptors) and smaller pretreatment tumor size. Pathologists should be aware of the clinical significance of HER2 IHC staining differences (particularly in the 3+ category) and HER2 FISH results in terms of predicting benefit from HER2-directed NAC and should emphasize these variables in a multidisciplinary setting to further clinicians' understanding of potential treatment responses.

## Figures and Tables

**Table 1:** ASCO/CAP guidelines categorize HER2 FISH results into 5 groups based on HER2/CEP17 ratio and average HER2 copy number.

Group	HER2/CEP17 ratio	Average HER2 copy number
1	≥2.0	≥4.0
2	≥2.0	<4.0
3	<2.0	≥6.0
4	<2.0	≥4.0 and <6.0
5	<2.0	<4.0

**Table 2:** Characteristics of patients with HER2+ breast cancer receiving neoadjuvant chemotherapy: evaluation of residual cancer burden in 151 patients.

	Total	RCB-0 (*n* = 64)		RCB-I (*n* = 32)		RCB-II (*n* = 47)		RCB-III (*n* = 8)		*p* values^∗^
	Mean (SE)	Mean	SE	Mean	SE	Mean	SE	Mean	SE	
Age	52.9 (11.8)	54.1	11.7	52.0	11.5	50.9	11.7	57.5	14.6	0.4700
Pretreatment tumor size	3.6 (2.3)	3.3	2.1	3.4	2.0	3.7	2.2	6.2	4.1	0.0517^†^
	*n*	*n*	%	*n*	%	*n*	%	*n*	%	
Pretreatment clinical stage										Na
1	18	12	18.8%	4	12.5%	2	4.3%	0	0.0%	
2	91	39	60.9%	18	56.3%	30	63.8%	4	50.0%	
3	42	13	20.3%	10	31.3%	15	31.9%	4	50.0%	
Tumor type										Na
IDC	134	59	92.2%	29	90.6%	40	85.1%	6	75.0%	
Micropapillary	11	4	6.3%	2	6.3%	3	6.4%	2	25.0%	
Mucinous	1	0	0.0%	0	0.0%	1	2.1%	0	0.0%	
Apocrine	1	0	0.0%	0	0.0%	1	2.1%	0	0.0%	
ILC	3	1	1.6%	1	3.1%	1	2.1%	0	0.0%	
Mixed IDC-ILC	1	0	0.0%	0	0.0%	1	2.1%	0	0.0%	
Tumor − infiltrating lymphocytes (TILs) > 50%										Na
	13	7	10.9%	2	6.3%	4	8.5%	0	0.0%	
Tumor grade										Na
I	1	0	0.0%	0	0.0%	1	2.1%	0	0.0%	
II	54	20	31.3%	13	40.6%	17	36.2%	4	50.0%	
III	96	44	68.8%	19	59.4%	29	61.7%	4	50.0%	
ER status										0.0027^‡^
Positive (10-100%)	88	28	43.8%	22	68.8%	31	66.0%	7	87.5%	
Negative (<10%)	63	36	56.3%	10	31.3%	16	34.0%	1	12.5%	
PgR status										0.3776
Positive (10-100%)	55	21	32.8%	11	34.4%	18	38.3%	5	62.5%	
Negative (<10%)	96	43	67.2%	21	65.6%	29	61.7%	3	37.5%	
Hormone sensitive (ER or PgR+)										0.02^‡^
Yes (10-100%)	89	29	45.3%	22	68.8%	31	66.0%	7	87.5%	
No (<10%)	62	35	54.7%	10	31.3%	16	34.0%	1	12.5%	
Multiple tumors on imaging (pretreatment)										<0.0001^‡^
Yes	22	1	1.6%	7	21.9%	10	21.3%	4	50.0%	
No	129	63	98.4%	25	78.1%	37	78.7%	4	50.0%	

^∗^Continuous variable *p* values are calculated using the Kruskal-Wallis test, and *p* values for the categorical variables are calculated using the Chi square or Fisher's exact test. ^†^Marginally significant. ^‡^Statistically significant.

**Table 3:** HER2 characteristics of HER2+ cases receiving neoadjuvant chemotherapy.

	Total	RCB-0 (*n* = 64)		RCB-I (*n* = 32)		RCB-II (*n* = 47)		RCB-III (*n* = 8)		*p* values^‡^
	Mean (SE)	Mean	SE	Mean	SE	Mean	SE	Mean	SE	
	*n*	*n*	%	*n*	%	*n*	%	*n*	%	
										<0.0001^§^
IHC category^∗^										
IHC 3+ 100%	102	51	81.0%	27	87.1%	22	47.8%	2	25.0%	
IHC 3+ <100%	24	8	12.7%	2	6.5%	11	23.9%	3	37.5%	
IHC 2+	22	4	6.3%	2	6.5%	13	28.3%	3	37.5%	<0.0001^§^
HER2 copies	19.7 (10.5)	22.5	8.8	22.6	9.8	15.5	11.5	9.8	6.3	0.0015^§^
HER2/CEP17 ratio	7.5 (5.9)	8.5	6.2	8.3	4.8	6.1	6	4.5	5.1	Na
FISH groups: HER2/CEP17 ratio; mean HER2 copies										
Group 1: ≥2.0; ≥4.0	134	60	93.8%	31	96.9%	38	80.9%	5	62.5%	
Subset 1A: ≥2.0; ≥4.0 to <6.0	9	1	1.6%	1	3.1%	7	14.9%	0	0.0%	
Subset 1B: ≥2.0; ≥6.0	125	59	92.2%	30	93.8%	31	66.0%	5	62.5%	
Group 2: ≥2.0; <4.0	1	0	0.0%	1	3.1%	0	0.0%	0	0.0%	
Group 3: <2.0; ≥6.0	7	2	3.1%	0	0.0%	3	6.4%	2	25.0%	
Group 4: <2.0; ≥4.0 to <6.0^†^	7	2	3.1%	0	0.0%	5	10.6%	0	0.0%	
Group 5: <2.0; <4.0^†^	2	0	0.0%	0	0.0%	1	2.1%	1	12.5%	

^∗^Not including two patients with IHC 0 and one IHC 3+ patient who does not have staining percent. ^†^One RCB-II case with heterogeneity. ^‡^Continuous variable *p* values are calculated using the Kruskal-Wallis test, and *p* values for the categorical variables are calculated using the Chi square or Fisher's exact test. ^§^Statistically significant.

**Table 4:** Univariate predictors of treatment response for HER2+ breast cancer patients receiving neoadjuvant chemotherapy.

Variable	OR (95% CL OR)	*p* value
Age	1.01 (0.99-1.04)	0.3758
HER2 absolute copy number	1.07 (1.03-1.1)	<0.0001
HER2/CEP17 ratio	1.07 (1.02-1.14)	0.0142
IHC_STAIN_PER		<0.0001^a^
3+ 100% vs. 2+	6.49 (2.60-16.25)	<0.0001
3+ <100% vs. 2+	1.88 (0.63-5.61)	0.2612
Clinical stage		0.0246^a^
1 vs. 3	4.78 (1.64-15.41)	0.0067
2 vs. 3	1.58 (0.81-3.12)	0.1797
Tumor grade biopsy		0.4041^a^
I vs. III	0.16 (0.0-5.44)	0.3294
II vs. III	0.73 (0.4-1.36)	0.3231
Pretreatment tumor size	0.85 (0.74-0.97)	0.0142
ER expression		
Negative (<10%)	2.03 (1.08-3.90)	0.0304
Positive (10-100%)	Ref.	
Hormone sensitive (ER or PgR+)		
Negative (<10%)	2.44 (1.32-4.53)	0.0046
Positive (10-100%)	Ref.	

Probabilities modeled are cumulated over the lower ordered RCB values. ^a^*p* value is associated with Type 3 analysis of effects.

**Table 5:** Multivariate models predicting residual cancer burden following neoadjuvant chemotherapy for HER2+ breast cancer.

Models	Characteristics	OR (95% CL OR)	*p* value
Model 1			
	Hormone sensitive (ER or PgR + (10-100%))		
	Negative vs. positive	2.85 (1.51-5.38)	0.0012
	HER2 absolute count	1.07 (1.02-1.14)	0.0107
	Pretreatment tumor size	0.83 (0.73-0.95)	0.0060
Model 2			
	Hormone sensitive (ER or PgR + (10-100%))		
	Negative vs. positive	2.83 (1.48-5.39)	0.0016
	HER2/CEP17 ratio	1.07 (1.03-1.1)	<.0001
	Pretreatment tumor size	0.83 (0.73-0.95)	0.0084
Model 3			
	Hormone sensitive (ER or PgR + (10-100%))		
	Negative vs. positive	3.19 (1.64-6.2)	0.0006
	IHC stain percentage		<0.0001^a^
	3+ 100% vs. 2+	7.59 (2.98-19.35)	<0.0001
	3+ <100% vs. 2+	2.24 (0.74-6.82)	0.1563
	Pretreatment tumor size	0.81 (0.71-0.93)	0.0028

Probabilities modeled are cumulated over the lower ordered RCB values. ^a^*p* value is associated with Type 3 analysis of effects.

## Data Availability

Data supporting the results of this study will be made available upon request. Please contact the corresponding author: karen.swenson2@allina.com.

## Additional Points

*Key Points*. (i) HER2 immunohistochemistry 3+ staining of less than 100% of sampled tumor cells shows weaker association with pCR and higher RCB scores following HER2-directed neoadjuvant chemotherapy compared to tumors with virtually 100% of cells exhibiting 3+ staining, despite all currently categorized as HER2 positive by CAP/ASCO. (ii) RCB-II and RCB-III tumors had lower HER2 FISH copies (*p* < 0.0001) and lower HER2/CEP17 ratios (*p* = 0.0015) compared to RCB-I and RCB-II cases. (iii) Estrogen receptor classification defined as ≥10% staining showed stronger association with lower RCB categories versus ≥1% staining by immunohistochemistry (IHC).
